# ‘What matters to you?’ Normative integration of an intervention to promote participation of older patients with multi-morbidity – a qualitative case study

**DOI:** 10.1186/s12913-021-06106-y

**Published:** 2021-02-04

**Authors:** Jannike Dyb Oksavik, Turid Aarseth, Marit Solbjør, Ralf Kirchhoff

**Affiliations:** 1 Department of Health Sciences, Ålesund, Faculty of Medicine and Health Sciences, Norwegian University of Science and Technology, Ålesund, Norway; 2 Faculty of Business Administration and Social Sciences, Molde University College, Specialized University in Logistics, Molde, Norway; 3 Department of Public Health and Nursing, Trondheim, Faculty of Medicine and Health Sciences, Norwegian University of Science and Technology, Trondheim, Norway

**Keywords:** Health care reform, Practice guideline, Patient participation, Patient care planning, Institutional logics, Normative integration, Delivery of health care, integrated, Vertical integration, Multimorbidity

## Abstract

**Background:**

Interventions in which individual older patients with multi-morbidity participate in formulating goals for their own care are being implemented in several countries. Successful service delivery requires normative integration by which values and goals for the intervention are shared between actors at macro-, meso- and micro-levels of health services. However, health services are influenced by multiple and different institutional logics, which are belief systems guiding actors’ cognitions and practices. This paper examines how distinct institutional logics materialize in justifications for patient participation within an intervention for patients with multi-morbidity, focusing on how variations in the institutional logics that prevail at different levels of health services affect vertical normative integration.

**Methods:**

This qualitative case study of normative integration spans three levels of Norwegian health services. The macro-level includes a white paper and a guideline which initiated the intervention. The meso-level includes strategy plans and intervention tools developed locally in four municipalities. Finally, the micro-level includes four focus group discussions among 24 health professionals and direct observations of ten care-planning meetings between health professionals and patients. The content analysis draws on seven institutional logics: professional, market, family, community, religious, state and corporate.

**Results:**

The particular institutional logics that justified patient participation varied between healthcare levels. Within the macro-level documents, seven logics justified patients’ freedom of choice and individualization of service delivery. At meso-level, the operationalization of the intervention into tools for clinical practice was dominated by a state logic valuing equal services for all patients and a medical professional logic in which patient participation meant deciding how to maintain patients’ physical abilities. At micro-level, these two logics were mixed with a corporate logic prioritizing cost-efficient service delivery.

**Conclusion:**

Normative integration is challenging to achieve. The number of institutional logics in play was reduced downwards through the three levels, and the goals behind the intervention shifted from individualization to standardization. The study broadens our understanding of the dynamic between institutional logics and of how multiple sets of norms co-exist and guide action. Knowledge of mechanisms by which normative justifications are put into practice is important to achieve normative integration of patient participation interventions.

**Supplementary Information:**

The online version contains supplementary material available at 10.1186/s12913-021-06106-y.

## Background

Facing aging populations, Western countries and their health authorities are looking for new ways to deliver health services according to patients’ needs. New practices go under various names, such as ‘integrated care,’ ‘integrated service delivery,’ or ‘joint working’ [1]. ‘Integration’ means combining organizational parts into a unified, synergistic whole [2]. Actors within the health system may have different views, interests and objectives [3]. The goal for patient care is not always shared, either across care settings or between health professionals and patients [4–6]. More than 60% of people over 65 have multi-morbidity, meaning they have two or more chronic diseases [4]. Patients with multi-morbidity often have complex health needs and functional decline and are dependent on long-term health care from several services [7]. In the past, individual older patients have been minimally involved in decisions about their care [8, 9]. A paradigm shift within the health system – towards letting patients’ values, needs and preferences direct health service delivery – is now required. Goal-oriented care is designed to engage patients in setting personal goals and to align care to attain these goals. This practice is assumed to increase patients’ health and self-management, improve quality of care and reduce costs [5, 6, 10–12]. Goal-oriented care is being included as an intervention within integrated care models and in clinical guidelines [4, 6, 13, 14]. However, in practice, integrated care proves difficult to accomplish [15–17].

Evidence indicates that normative integration ensures collaborative processes within the health system [2, 18]. Normative integration means that actors have a common frame of reference and shared values and goals for service delivery [16, 19]. Values and goals must span the micro- (professional), meso- (municipal/organizational) and macro- (national/government) levels of health services. Vertical integration through these levels is a condition for implementation and accomplishment of integrated service delivery [13, 20, 21]. So far, research shows that normative integration of interventions is negligible, and research into how normative integration functions is itself sparse [19–21]. Normative drivers may facilitate or constrain patient participation, and empirical studies of how values connect to behavior are called for [13, 15, 21]. To reduce this knowledge gap, the present paper investigates normative integration from a novel perspective, connecting values with actions by focusing on how actors at different healthcare levels are guided by particular institutional logics. Institutional logics are societal belief systems which provide actors with frames of reference that precondition their sensemaking choices [22]. The lens of institutional logics is here applied to an initiative meant to enable goal-oriented care in Norwegian municipal health services.

### The institutional logics perspective

This perspective understands individual and organizational behavior within the societal and institutional context [23]. The viewpoint developed out of a critique of the ways in which institutional analysis ignored issues of change and the effects of human agency [24]. Institutional logics considers ‘the socially constructed, historical patterns of cultural symbols and material practices, assumptions, values and beliefs by which individuals produce and reproduce their material subsistence, organize time and space, and provide meaning to their daily activity’ [25] p.51.

Studies have typically examined institutional logics by using typologies, and one of the most influential typology is presented by Thornton, Ocasio and Lounsbury [22, 23, 25]. According to these authors, institutional logics are embedded in seven societal institutional orders which, to varying extents, govern actors and fields: the family, the community, religion, the state, the market, the profession and the corporation [25]. These orders highlight the interplay between individuals, organizations and institutions from macro- to micro-level and vice versa [25]. The logics they embody establish core principles according to which actors organize activities and channel interests. Logics shape action [25], and actors in turn draw on different institutional logics for meaning and motive. Actors can manipulate and elaborate different logics for their own advantage and to change social relations [23].

Institutional logics have regulative, normative and cognitive dimensions. The normative dimension is connected to actors’ values and goals [25, 26] and can illuminate normative integration between healthcare levels involved in the patient participation intervention under consideration in this study. Values are conceptions of what is preferred or desirable, and values supply standards according to which existing structures or behavior can be assessed. Norms specify how things should be done; they define legitimate means of pursuing valued goals. Institutional logics constitute various justifications for why goals should be pursued in health services [26], see Table 1.

**Table 1:** How the basis of norms differs between the seven institutional logics

Logic	
The professional logic	..entails autonomous judgment based on specialist knowledge. Norms are professionally developed and controlled by others in the profession [25, 27].
The corporate logic	..allows actors to achieve organizational goals through reproduction and efficiency by gaining authority over others [28]. Routines and administrative control of managers determine norms and procedures [27, 29].
The market logic	..lets consumer preferences, satisfaction and choice determine norms within the context of a broader market [25, 27, 30].
The community logic	..means that group membership gives a sense of belonging, maintained through reciprocities, trust and commitment to shared values. This supplies local norms for organizational practices [25, 31].
The state logic	..involves securing social and political order [32]. The government takes direct responsibility for health care and determines appropriate quality standards for care [27]. The basis of norms is citizenship in a nation [25].
The family logic	..involves fellowship and unconditional loyalty to family members and their needs [24]. Norms are related to membership in household [25].
The religious logic	..emphasizes the importance of faith and sacredness. The basis of norms is membership in a congregation [25].

While the literature suggests that individuals and organizations are confronted with diverse normative requirements and multiple institutional logics, studies of health services have typically focused on two to three competing logics [25, 26]. The professional logic has traditionally dominated research on health services; however, some studies shift the emphasis toward corporate and market logics [33, 26]. Health professionals may experience incompatibility of values between the professional logic and corporate principles, as business-based models of health care in which governance structures have been changed to increase efficiency and ‘do more with less’ [34] and an emphasis on cost-effective treatment and using the lowest-cost provider compromise patient participation [35, 36]. The logics of religion and family are currently underexplored in relation to health services [37]. Few studies have examined multiple logics between levels of health services [38].

When multiple logics are in play, they may facilitate or constrain action [33, 27]. The constellation of institutional logics describes the relationship among multiple logics at a given time. If increase in the strength of one logic does not correspondingly decrease the strength of another, the constellation is cooperative. In a competitive constellation, increases in the strength of one or several logics correspond to a decrease in the strength of another. Nondominant logics carry less force in guiding behavior [25, 27]. Few studies have explored how multiple institutional logics influence health services for older patients with multi-morbidity. This relates to the call for research on normative integration, exploring whether actors share goals and whether cultural norms support formal protocols [2].

### The case: vertical integration of an intervention involving patient goal setting

The Norwegian case is typical of a paradigm shift seen in a number of high-income countries over the past decade toward health policies designed to increase patient participation and health services which implement integrated care models [4, 13, 14, 39]. The case is a specific goal-setting intervention, examined through analysis of the health policy that triggered the intervention, a clinical guideline, intervention tools and health professionals’ practices. We do not evaluate the implementation process; rather, we focus on the justifications offered for increasing patient participation and the institutional logics in play in those justifications in order to understand whether and how vertical normative integration occurs between health service levels.

The case is based on a health reform for Norwegian municipalities proposed in the white paper “A full life - all your life A Quality Reform for Older Persons” [40]. This white paper and an accompanying national guideline are key instruments for increasing patient participation [40, 41]. The target group of the reform is actors who deliver health services for people over 65 years who live at home or in institutions [40]. The guideline for follow-up with patients with multi-morbidity has a similar objective [41].

The Norwegian state is social democratic and universalist [42]. Services for older people are broadly accessible and are primarily financed, organized and delivered by public entities in the municipalities [43]. These entities include facilities for rehabilitation and long-term care, which takes place in community hospitals for rehabilitation, in nursing homes, or in patients’ homes. Patients can also receive time-limited and intensive rehabilitation service in their homes.

The intervention entails that when individual patients are allocated health care services by their municipalities, health professionals ask each patient ‘What matters to you?’ to enable patient participation in decisions about how these services should be delivered [5, 41]. A goal for care is formulated and documented with the understanding that patients and health professionals will work together towards this goal. This planning of care with patients occurs either in conversations with one health professional or during interprofessional meetings. Health professionals include nurses, auxiliary nurses, physicians, physiotherapists and occupational therapists. Patients’ goals may relate to reducing symptoms or improving physical functioning or well-being; goals can also have social dimensions or be related to life values [6, 41].

Integrated service delivery often takes place in collaborative networks spanning levels [21]. This is a form of collaboration based on social commitment rather than a formal hierarchy of the kind that might be seen under traditional top-down governance, based upon legal duties or market-based contracts [44]. Within newer forms of governance, guidelines are issued from the macro-level, but each level determines how to carry out its responsibilities. The white paper and the guideline offer normative recommendations, which may be adjusted to local contexts by each municipality [40, 41]. Actors across levels in Norwegian municipalities can participate in a national collaborative quality improvement network for integrated care, in which the intervention is proposed [45]. The intervention is operationalized at the meso-level through the development of tools which are then used by health professionals carrying out the intervention at the micro-level. A dynamic interaction ideally occurs between the policy level and micro-level norms and behaviors [2]. Actors need a shared vision of why interventions should be carried out [17]. However, little is known about how institutional logics influence actors’ justifications for encouraging patient participation. To illuminate vertical normative integration within Norwegian municipal health services for older patients with multi-morbidity, we ask:

1. In what way are normative justifications for patient participation connected to different institutional logics?
2. How do the constellations of institutional logics vary between the macro-, meso-, and micro-levels of health services?

## Methods

### Design

**Fig. 1:**
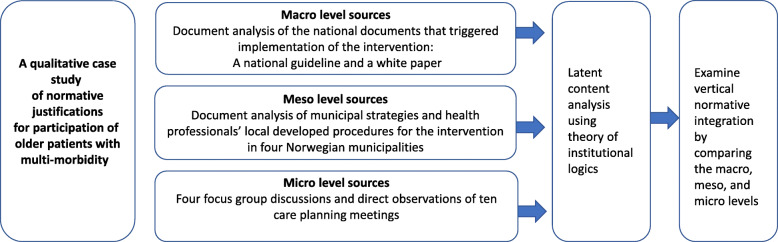
Overview of the research process and sources of data

A qualitative case study method allows the examination of the intervention and the institutional logics at work. This study includes three embedded units of analysis (macro-, meso- and micro- levels) [25, 46]. The institutional logics perspective is grounded in social constructivism, in which beliefs and norms held by institutional actors are seen as socially constructed and shared [25]. The case study allowed us to observe these shared norms within the actors’ context and to triangulate data to achieve ‘thick’ descriptions [46–48]. Institutional logics are captured in language, practices and materials [49]. Thus, to achieve our aims, we combine analysis of the documents that triggered implementation of the intervention, focus group discussions and direct observations of meetings between patients and health professionals [46, 48, 50]. Figure 1 gives an overview of the research process. All sources of data were analyzed using latent content analysis, which seeks the underlying meaning of the text [51]. We associate this underlying meaning with relevant institutional logics [25]. In line with constructivist approaches, descriptions produced in this study and results obtained are considered to be interpretations influenced by the researchers and their context [48].

### Sample

To investigate normative integration, we selected data which contained normative statements about patient participation and covered different actors’ perspectives. We purposively chose four municipalities which had implemented the intervention and carried it out as described in the introduction to this article. These municipalities participated in a national collaborative quality improvement network for integrated care [45] and implemented the intervention 6–12 months prior to our data collection. The municipalities are located in Western Norway. Two are rural, with 2000–3000 inhabitants each, while two are cities with 40,000 and 70,000 inhabitants.

### Documents

Sampling of documents was purposive: We selected all macro-level documents designed to be used by all levels of the health system which contained guidance and normative recommendations for carrying out the intervention. The governmental white paper “A full life - all your life A Quality Reform for Older Persons” describes how ‘What matters to you?’ should form the basis of service delivery [40]. Institutional logics tend to materialize in white papers, which are, among other things, attempts to govern meanings about what should be done, and which exemplify the dominant official narratives of their times [52]. The clinical guideline for follow-up of persons with complex needs is the first Norwegian guideline describing integrated care for older patients with multi-morbidity [41].

At meso-level, municipal strategies for eliciting the participation of individual patients were extracted from the municipalities’ web pages. We used search terms that covered care pathways for older patients with chronic diseases, patient participation at the individual level and the phrase ‘What matters to you?’. We included all text concerning these matters, which was amounted to 500–1000 words per municipality. To protect the anonymity of study participants, we do not refer to these webpages, as doing so would identify the particular municipalities. The municipal strategies were included to examine whether the intervention was included in prevailing policy within each municipality. To capture the institutional logics being applied in local materials [25], we also considered tools used by health professionals to enable the intervention, such as care pathway checklists.

### Focus groups

To examine health professionals’ justifications for patient participation, we arranged one focus group discussion [50] in each municipality, convening health professionals from multiple sites. One participant with no health education was included because health education is not required for all employees in Norwegian municipal health services. Managers or municipal workers issued invitations, either in person or by email, to 27 health professionals who worked in clinical settings and had experience with the intervention.

### Observations

Ten care-planning meetings in which the intervention was carried out were observed. Eligible patients had two or more chronic diseases and a current need for more health services. The intervention was a component of municipalities’ integrated care pathways for older patients with multi-morbidity. The pathway was mainly used for patients over 80 but could be used for younger patients in rehabilitation wards. We aimed for a purposive sample of meetings, representing different kinds of wards and different stages of the care pathway. Health professionals recruited patients, and the meetings we observed were planned independently of this study. Patients in the end of life-phase or with cognitive impairment were excluded.

### Data collection

#### Documents

In August 2019, we retrieved the national guideline [41], the white paper [40] and the municipal strategies for health services from the internet. We thoroughly read the white paper “A full life - all your life A Quality Reform for Older Persons” [40]. Then, we extracted the chapters describing patient participation: Chapter 1 (‘Goals and target group’), Chapter 7 (‘Health care’) and Chapter 8 (‘Coherence’). From the guideline [41], approximately 20 of 63 pages were excluded because they referred to younger patients or other organizational work tasks. The meso-level care pathway checklists and tools developed for health professionals who carried out the intervention were identified by, and collected from, health professionals in each municipality from October 2018 to December 2019.

#### Focus groups

The focus group discussions [50] occurred from September 2018 to February 2019. Each of the four groups consisted of 5–7 participants. They took place without interruption in meeting rooms at participants’ workplaces. A semi-structured interview guide prompted health professionals to describe and discuss goal-setting situations they had experienced in care planning with older patients with multi-morbidity. The interview guide was developed by the first author to elicit information about health professionals’ patient participation practices, that is, what they had done in specific situations. We asked for their justifications for why and when they could (or could not) act upon patients’ preferences. This allowed us to examine the salient institutional logics governing their justifications, even though we did not explicitly ask about institutional logics (see Additional file 1 for the interview guide). The discussions lasted an average of 90 min.

#### Observations

The first author conducted direct observations of care-planning meetings in which she attempted to assume a neutral role [48]. She engaged in small talk before and after meetings but did not speak during the meetings. An observation guide developed by the first author was filled out about the setting and patient participation in goal setting (see Additional file 1). The health professionals were told that the aim was to observe the intervention; however, they were not told in detail which aspects were being observed. The observations were made from October 2018 to December 2019. Except for one meeting, all interviews and observations were audio-recorded and transcribed verbatim. The first author previously was a hospital nurse for older patients with multi-morbidity. The transcripts allowed the co-authors, who have different backgrounds, to interpret the material. Field notes were written immediately after each observation. The meeting agendas were similar across these settings; therefore, ten meetings were sufficient to assess how the intervention was carried out.

### Analysis

We started by analyzing the macro-, meso- and micro- levels separately. Subsequently, we looked at the whole of the case and compared the three levels [46]. All sources of data were analyzed using latent content analysis. The analysis process comprised four steps: decontextualization, recontextualization, categorization and compilation [51]. In the decontextualization phase, theory and definitions of the seven institutional logics (see Table 1 in the introduction) gave guidance for initial codes of all documents and transcripts [51]. To identify normative justifications for patient participation, we coded statements such as ‘health professionals/the services should…’ and ‘the goal is to … ’ and statements of why participation was important. JDO and RK separately coded the data and regularly discussed coding with each other and the co-authors. It was essential through the analysis to interpret the whole of all texts to understand the case. Texts which could be coded to two logics were discussed to reach consensus. During the recontextualization phase, meaning units and text extracts were inserted in tables. Text not relevant to the research aim was excluded. In the categorization phase, we found properties of the seven institutional logics and the constellations they constituted at each level. The logics in the documents were classified as weak or strong depending on how frequently they appeared, how thoroughly they were described and the normative words (health professionals ‘should’ or ‘have to’ vs. ‘can’, e.g.) with which they were associated. At micro-level, the strength of logics was determined by how frequently they appeared and whether one logic seemed to prevail over another in guiding decision-making with patients [25]. Logics neither mentioned by participants nor found in materials were coded as ‘did not appear’.

The ways in which the institutional logics were applied to patient participation were coded according to justifications for, and aspects to consider, when encouraging patients to participate [35]. Table 2 gives an example from the coding process.

**Table 2:** Example from coding of the state logic

Data	Units of meaning	Subcategories	Theme
**Extract from white paper:** “Older persons should feel valued, seen and be able to participate in decisions which involve them. They should have the opportunity to live at home as long as possible, and receive support to master their everyday lives, regardless of illness or functional impairment” [20] p.121.	Seen as individuals. Supported mastery, with focus on living everyday life. Treatment at home is the norm. Support from health system. Reduced health no obstacle.	The state determines quality standards for care and role of the health system [27]. The state expects older persons to participate in order to master life at home.	Individualized service delivery

In transcripts from micro-level, we examined how active patients were expected to be in goal setting: from being excluded, to being informed about decisions, to being invited to express their preferences or collaborate in goal setting [6, 8, 35].

In the compilation phase, we examined the constellation of logics across the macro-, meso- and micro-levels, interpreting the constellation of logics at each level as either facilitating or constraining patient participation. Finally, to assess normative integration, we compared the constellation of logics vertically across health care levels [2, 27, 53]. The software NVivo 12 Pro qualitative data analysis software (Melbourne, Australia: QSR International Pty Ltd., 2018) supported the analysis. The Norwegian text extracts were translated to English by a translator after the analysis.

## Results

### Participants

Three invitees to the focus groups did not attend due to illness. Participants included four head nurses in nursing homes, one head nurse in home care services, seven nurses, one caseworker, three auxiliary nurses, two occupational therapists, four physical therapists, one physician and one person without health education. Two of the focus groups has one male participant each. Five participants were between 20 and 30 years old, 10 were between 30 and 40, four were between 40 and 50 and five were 50–65 years old. In the observed care-planning meetings, the mean age of the ten patients was 88 (with a range from 62 to 98); two were men and eight were women. All patients had multi-morbidity. Plans were made for eight patients to go home and two to receive long-term care in institutions. Four of the meetings were carried out by a nurse, and these lasted an average of 32.5 min. Six meetings were carried out by an inter-professional team, and these lasted an average of 47 min. Relatives participated in seven of the meetings.

This section proceeds by firstly explaining how normative justifications for patient participation were connected to different institutional logics at the macro-, meso- and micro- levels of health services. Secondly, it discusses how the constellations of institutional logics vary vertically through the levels of health services.

### The macro-level documents: multiple cooperative logics

All seven institutional logics from the theoretically based typology [25] appeared in the white paper and guideline. The institutional logics were associated with distinct views of patients and particular justifications for increasing patient participation. These justifications aimed to allow patients to become drivers of their own lives. The guideline called for a paradigm shift towards more patient participation, arguing that health services should take a holistic approach by allowing multiple areas of individual patients’ lives to be acknowledged in the formulation of plans for service delivery [41] p.15-16. A main point was to shift from a traditional professional mindset in which medical knowledge guides care planning.

The question [What matters to you?] also poses a challenge for the traditional professional role as an ‘expert’. It’s about aiding the person in finding optimal individual solutions rather than giving fast answers. (guideline, [41] p.16).

Both the guideline and the white paper described a mode of service delivery not dominated by health professionals’ medical judgments. A shift towards patient empowerment, including the further development of professional skills such as listening and transferring power to patients, was emphasized. This gave the professional logic a more person-centered emphasis. The white paper invited actors in health services to think differently about older people, who were presented as members of a local community, having individual life stories and personal interests and activities to be included in care planning.

A person-centered approach involves (…) seeking to understand the world from the individual’s perspective and accommodate his or her social and psychological needs. The residents’ life stories, values and preferences should form the foundation for formulating and carrying out the services. (white paper, [40] p.149–150).

Moreover, care planning could support older people to master the tasks of everyday life and remain involved in their communities despite functional decline [41]. The white paper pointed out that people prefer service delivery at home. Both the guideline and the white paper stipulated that services should be delivered in a family and social-network perspective. These recommendations reflected perceptions of older persons as family members in their community, perceptions which indicate an interplay between the family and community logic. Hence, relatives with a caregiver burden should also be given greater opportunities to participate.

The services should make room for participation of the patient’s loved ones, family, and network according to the patient’s wish. (guideline, [41] p.16).

The documents emphasized that to achieve these goals, services should not be planned according to standardized routines of service delivery. Moreover, the white paper drew on principles from a market logic in descriptions of older patients as consumers with freedom of choice.

‘Live Your Whole Life’ is a reform intended to provide a greater freedom to choose. It should give each individual better opportunities to choose service providers (who), be involved in the content of the services provided (what), determine the manner in which services are provided (how), and the time and place for the provision of services (where and when). (white paper [40] p.10.

Giving patients freedom of choice would be beneficial in the context of the logics of both market and corporation: patient participation was justified by its benefits for patient health. Supporting patients to attain control of their own lives and maintain their health would subsequently lead care processes to become more efficient.

Several of the suggested solutions can increase the efficiency of the services and decrease the need for help for the elderly in the long term. (white paper [40] p.175.

Moreover, the white paper requested that health professionals address individual patients’ faith, philosophical practices or need to discuss existential questions. However, few other statements associated with a religious logic were present, indicating that this logic was weak. Thus, the content of the white paper and guideline modified the professional logic and strengthened the logics of community, family, market and religion, which were all associated with individualized service delivery that would take social and psychosocial dimensions of patients’ lives into account.

The logic of the state provided a broader societal justification for patient participation. Including older citizens as co-producers of service delivery would increase society’s capacity to handle the growing population of older people. Older peoples’ functional abilities often decline, so they were expected to take an active role by setting goals to maintain health.

For health and care services, this will mean, among other things, being more resource-oriented and placing greater emphasis on proactivity, early intervention, prevention and everyday coping, often based on the basic question: What is important to you? Most people want to participate and manage themselves for as long as possible, and that is also the best for the community and future sustainability. (white paper [40] p.53.

One goal of the intervention was to provide equal treatment and reduce geographical inequalities. Tools were suggested to improve quality-of-care pathways, such as the Patient-Specific Function Scale [54] and checklists. This suggests that the intervention was governed by the logic of the state. Equal treatment, however, introduced ambiguities in the context of the intervention to individualize services, because the guideline described standardization as a complex task due to the inherent complexity in patients’ multiple diseases and their need to make use of several services within fragmented organizations.

In follow-up of patients (...) there are limits to how much it can be standardized. (guideline [41] p.7.

In sum, the white paper and the guideline reflected the intention of reducing competition between different logics, e.g. dominant professional logic and the hitherto weaker logic of family. The suggestions within the documents for increasing patient participation by including several areas of patients’ lives can be associated with an effort to give equal value to multiple institutional logics, which is a move towards a cooperative constellation of logics. The constellation of logics at this level, in sum, constituted the following norm for patient participation: Health services enable patients to be the drivers of their own lives and live full, independent lives in their communities with support from health authorities. To achieve this, local solutions in the municipalities were called for.

### The meso-level documents: From a constellation of multiple cooperative logics to two dominant logics

The move articulated at the macro-level towards a cooperative constellation of logics was manifested in municipal strategies for individual patient participation, as described on public municipal websites. The municipalities described ‘What matters to you?’ as an individualized basis for service delivery, often referring to the white paper. Methods to incorporate patient participation in health services were described in general terms rather than in terms of detailed practices.

There should be a focus on user participation, and everyone involved must ensure user participation when making decisions. (Strategy plan on website, Municipality 2, rural).

To guide health professionals’ practice, each ward had written tools for goal setting with patients. These had been developed by health professionals, often ward managers, or adopted from other municipalities.

The tools used differed between municipalities (Table 3), but in general they had two main functions. The first function was to plan how patients could manage at home.

This procedure is aimed at follow-up of patients discharged from the hospital who need a plan going forward for what steps need to be taken for the sake of continuity in their care pathway. Meeting agenda: Map out and plan future care needs with returning home as the end goal. (Written agenda for interprofessional meetings with patients, Municipality 3, rural).

**Table 3:** Intervention tools developed at meso level

	Municipalities			
Intervention tools	1	2	3	4
A ‘What matters to you?’ questionnaire based on the Patient-Specific Functioning Scale [54], used to set goals for physical rehabilitation.	**x**			**x**
Pocket cards for health professionals, with three questions to elicit patients’ rehabilitation goals.	**x**			**x**
Form to fill in medical information about new patients, with ‘What matters to you?’ as one of approx. 15 items.	**x**	**x**		
Written agendas for care-planning meetings, in which elicitation of patients’ goals for their care pathways was one component			**x**	**x**
One-to-two-page care pathway checklists with an open-ended question ‘What matters to you?’	**x**	**x**	**x**	**x**

Secondly, the tools were used to obtain an overview of medical information and physical function.

*The meeting agenda: a plan for the road ahead.*

What was your condition before your last hospitalization? How are you now? How would you like your condition to be? What needs to happen for you to achieve that? (Written agenda for care-planning meeting. Municipality 4, city).

These tools had the effect of limiting the areas within which patients could set goals. From macro- to meso-level, the professional logic shifted from empowering patients to master what mattered to them to letting medical knowledge guide formulation of goals for service delivery. The professions followed medical standards by including “What matters to you?” as one of many items on existing forms used to map medical information about patients. Moreover, the fact that the question was integrated into care pathway checklists for patients with multi-morbidity could be interpreted as expressing a state logic emphasizing standardization and bureaucracy, in which as much focus is given to asking all patients as is given to their answers. The focus group discussions at micro-level reflected such ideas. Moreover, a weak corporate logic was also in play, because these checklists served the function of facilitating managers’ control by process-evaluation of whether professionals at the ward had asked all patients the required questions. What is more, through these tools, patients’ goals were pre-defined in terms of going home. In this way, the tools restricted which health services and areas of patients’ lives services could focus on.

The texts at meso-level contained fewer logics than at the macro-level. We found few justifications for patient participation rooted in a logic of family. The tools had no text that prescribed that health professionals should solicit relatives’ preferences in decision making or attend to the patient’s position in a family. The logic of community was weak as well; the actors who developed the tools apparently less considered patients’ positions or participation in a community, life stories, or interests and hobbies. The market logic which, at macro-level, emphasized individual choice did not appear here. The tools in use did not encourage patients to choose times or places for service delivery, nor to determine which health professionals to involve or how the allocated services should be delivered. Attending to patients’ religion was briefly mentioned in one of the municipalities’ strategy plans and in one of the tools. Moreover, the tools indicated that a corporate logic dominated over a logic of family, since the help text for health professionals in the tools described family members as helpers in patients’ management at home and, thus, contributors in service delivery.

Ask the patient: ‘Do you have family or friends who assist you with your everyday chores or activities?’ Ask what patients’ relatives can do to help patients achieve their goals. (Tool for health professionals, pocket card. Municipality 4, city).

The cooperative constellation of multiple logics found at the macro-level broke down in the written tools in use at meso-level: several logics became weaker as the intervention was operationalized and adjusted to the health services’ existing structures. The logics of profession and the state dominated in determining how questions to patients were formulated. The tools often had sparse guidance text informing health professionals of how, why, and with what consequences they should ask patients ‘What matters to you?’. Overall, the constellation of logics in operation at the meso-level constituted the following norm for patient participation: All patients should participate in setting goals about how to manage to live at home.

### The micro-level practices: three dominating logics

This level includes focus groups with health professionals and observations of care planning meetings. The emergent theme from the focus groups was that multiple logics in the field created a tension between individualized and standardized service delivery. Health professionals perceived their practices to have changed because of the intervention – they felt more aware of patients’ preferences. Health professionals reflected the white paper’s view of the professional logic, to empower patients to find individual solutions.

P2: I’m thinking that since we started asking “What is important to you?” it has maybe become easier to focus on their goals. Before, we might have been the ones saying: “Yeah, maybe it is important for you to use a walker,” rather than “Can I use a walker?” But now it is more up to them to say that, before we come in, at least. And I think they see that as a positive thing.

P1: It has been a gradual shift, from a national health service with a very paternalistic approach where we know what is best for you. Now it’s more like we are more...we are on their team. (P1: Head nurse P2: Occupational therapist, both at rehabilitation ward. Focus group in municipality 1, city).

There were, however, some discrepancies between the comments made in focus groups and what we observed in meetings regarding the extent to which health professionals actually explored patients’ goals: The allocation of time in patient meetings indicated that the medical aspect of the professional logic was stronger: approximately four minutes out of an hour were devoted to conversation about ‘What matters to you?’ and patients’ personal goals, while most of the time was spent by health professionals collecting medical information and setting physical goals for patients. The focus group discussions revealed that this professional logic dominated the market logic of choice described at macro-level: Patient participation was not represented as freedom of choice regarding service delivery. Often, negotiations relating to autonomy occurred.

Yes, oh yes, but the question is what is important to the patient. If he says ‘It’s important I get to rest before I go home,’ should we still listen to the patient’s wish, should we work according to the patient’s wish or should we work against the patient’s wish ‘You have to exercise, you have to go through rehabilitation and make an effort,’ and... it’s not easy. (Nurse in focus group. Municipality 3, rural).

The goal-setting tools affected the structure and agenda of the ten meetings between health professionals and patients. Health professionals formulated the question ‘What matters to you?’ nearly verbatim as stipulated by the tools. Thus, the logics of state and profession written into the tools at the meso-level continued to dominate.

The focus group discussions revealed that the logic of state was dominant because patient participation in goal setting was mostly a means of planning how patients would manage at home. In addition, one clearly expressed norm was to distribute services equally to all patients out of the municipalities’ standard set of services. Sometimes, a mismatch occurred between the services available in a municipality and patients’ preferences and what fit into their routines. In such situations, health professionals adhered to standardized care and valued a cooperative patient role. This bureaucratic approach can be associated with blended logics, specifically the value placed by the state on equal treatment and the managerial principles of cost-efficient care processes stipulated within corporate logic.

P4: Because we home care nurses don’t have the time or space or capacity to treat people differently. We don’t care whether you’re a king or a hatter. You will get the help you need, what we can provide you with, what you need and what is important to you. (…) There is equal treatment. It doesn’t matter who you are. You will get what you need.

P3: Same with us, too. (P4: nurse home care services, P3: Occupational therapist in reablement services. Focus group, municipality 1, city).

Moreover, the logic of family appeared to be suppressed by the dominating logics at this level. Health professionals frequently undertook negotiations with relatives. In focus group discussions of such situations, they appeared to be influenced by a cost-efficiency mindset in which relatives could assist in health service delivery (corporate logic); they perceived relatives as lacking the skills to assess patients’ needs (professional logic) and often provided usual care instead of following relatives’ preferences (state logic). All three of these three logics that dominated at the micro-level are bureaucratic, and they overpowered the logics associated with individualized services, namely those of family and community, which would have prioritized attending to patients’ families or life stories, and the market logic of personal choice. A religious logic did not appear at the micro-level.

Our observations of how the tools developed at meso-level were used at micro-level and of the care-planning meetings and focus groups all indicated that health professionals adhered to three logics simultaneously in decisions about service delivery: health professionals’ medical standards for how to handle symptoms of patients with multi-morbidity (professional logic), a state logic of bureaucracy, and a cost-efficiency principle imposed by a corporate logic. Other logics hardly appeared, even though there were a few instances of invitations to include the patient’s individual goal in a more open way:

Meeting leader (head nurse): ‘What is important to you?’

Patient does not respond; she continues: ‘What do you value right now? What should we be keeping in mind? Have you set any goals? Any wishes that could make the road ahead easier?’

Patient: ‘Difficult question to answer.’

Meeting leader: ‘Well, you can always think about it. ( …). Is there something you are excited about or look forward to when you come home?’

Patient: ‘Play the pipe organ. And if I can get hold of some drawing materials.’

Meeting leader: ‘You draw?’

Patient: ‘No, I want to.’

(interprofessional meeting for an older man with multi-morbidity, rural municipality)

The constellation of logics at this level, in sum, constituted the following norm for patient participation: All patients should participate in making care processes efficient by setting goals to manage life at home.

### Differences between health service levels

Within the normative integration process, the professional and state logics were strong and transcended levels. The relationship between these logics helped them strengthen each other and materialize bureaucratically. There were subtle nuances in the normative justifications for patient participation: Participation as conceived at macro-level entailed enabling older persons to live independently in the community as a matter of right, while participation at lower levels was conceived in terms of allowing patients to contribute to care planning that favored efficient care processes directed toward the goal of going home. The intervention at macro-level attempted to shift the content of the professional logic, but at micro-level its traditional medical focus in decision-making persisted.

**Fig. 2:**
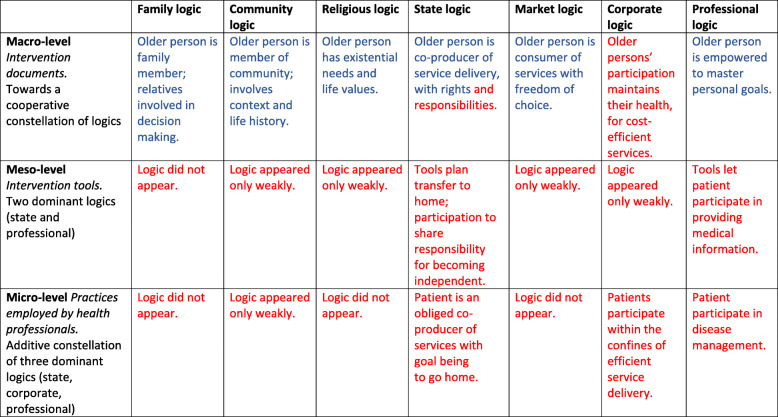
Institutional logics of patient participation between levels of health services

Figure 2 summarizes the main findings. The normative justifications supplied within each logic differed between levels. The columns show how each of the seven institutional logics materialized at each level. At meso- and micro-level, multiple logics appeared only weakly, and three logics vanished. The rows show how the constellations of logics differed between levels. At macro-level multiple logics were in play, while the meso- and micro-levels were more similar to one another, applying fewer distinct logics. Logics with blue letters are associated with individualized service delivery and those with red letters with standardized service delivery.

## Discussion

The results from our study show how normative justifications for patient participation are connected to different institutional logics and how constellations of logics vary between health service levels. The macro-level white paper and guideline were associated with multiple cooperative logics, through which several areas of patients’ lives were conceived as relevant to goal setting (Fig. 2). Patient participation was justified by the idea that health services should make it possible for patients be the drivers of their own lives and live full, independent lives in their communities. Throughout the documents, we found attempted to strengthen the logics—family, community and religious logics and a market logic of choice—that promote individualization and to shift the professional logic from a medical to a person-centered conception (Fig. 2). However, the constellations of logics at the lower levels reflected an imperative to standardization rather than individualization. The meso-level operationalization of tools for practice reflected a state logic which focused bureaucratically on the idea that all patients should set goals for independence at home, while a professional logic specified medical means of achieving this end. Finally, health professionals at the micro-level adhered to three logics simultaneously in goal setting with patients: a professional logic focusing on medical goals, a state logic emphasizing the importance of equal treatment and a corporate logic prioritizing cost-efficient care processes in which patients set goals to manage life at home.

Our discussion will be centered around how the applied institutional logics formed different intervention goals. The normative justifications for patient participation representing either individualization or standardization is an overall finding. Individualization proposed at macro level is comprehensive and consistent with ideals of goal-oriented care, which attend to individual patients’ values, needs and preferences and take a holistic view of patients, their families and their contexts [6, 7, 13, 39]. The Norwegian health services are based on integrated care models which recommend going from traditional to individualized service delivery, by for example supporting patients by including their relatives, community and informal social network [7, 14]. Institutional logics of family and community could have been expected to appear more strongly at lower levels in our study. Weak appearance of the market logic may be explained by the fact that in Norwegian municipalities, the public health services are not strictly market-regulated because the municipal services often are the sole supplier [43]. However, the standardized approach that we found is in line with other studies. The professional and state logics were found to dominate the process of implementation of multi-professional chronic care [53]. Moreover, health professionals’ adherence to the medical professional logic and efficiency proposed by a corporate logic has been found in other contexts as well [34, 35]. One systematic review found that efficiency is a value that transcends the various levels of integrated services [3] and which can be associated with standardization. This co-existence of principles of individualization with those of standardization which we found, and the conflict between them, is consistent with studies of competing institutional logics and of the challenges faced by health professionals in balancing these principles when encouraging older patients to participate in decisions about service delivery [55, 56].

Our findings of less person-centered approaches opposed those reported in Zonneveld and colleagues’ Delphi study of values in integrated services, which identified empowerment and person-centeredness as the most important values at the micro-level and the least important at the macro-level [21]. We found that health professionals claimed to value person-centered care; however, this value was not fully reflected in their practices. Few studies have examined the connection between values and action in integrated health services [21]. A possible explanation for our findings of discrepancies between values and actions at micro-level could be that actors’ values do not necessarily guide actions. Copeland [57] points out that it is not always best to follow guidelines, because the decisions health professionals make about whether to take a moral action must be weighed up in terms of potential consequences and utilities. Guidelines are not rules to be enforced in practice without considering contextual circumstances [57]. Other obligations in the situation, and especially dominating logics, could have led the health professionals to less often discuss individual patients’ preferences, even though the guideline requires it. Actors in health services can also manipulate and elaborate various logics to their own advantage [23]. The normative pressure may come from the professions themselves. The professional logic has traditionally been strong in decision-making [25, 27, 35]. The medical complexity of caring for older patients with several diseases [7] could lead to the tendency to adopt a standardized approach. Complexity in professional work is a task characteristic associated with standardization, because standardization maintains care pathway enactments by professionals [58].

Furthermore, institutional logics inform normative integration because constellations of logics form structures between health service levels which can enable or constrain action [53]. Logics can be a toolkit or a set of rule-like structures [25]. We interpreted the white paper as an attempt to make multiple logics equivalent and cooperative. This would provide health professionals with multiple available logics and increase their ability to exercise discretion and individualize service delivery [27]. However, at the micro-level, three logics had to be adhered to simultaneously in decision making. This situation allowed less space for health professionals’ creativity and fewer opportunities to exert discretion like what may be beneficial in goal-oriented care, because the work task had to satisfy the demands of more than one logic simultaneously [53]. Actors’ behavior is, at least in part, driven by the normative pressures of achieving the goals of the logics according to which they work [25]. Moreover, the dominant logics at the micro-level were bureaucratic, and bureaucratic logics are known to be less generative of change [25]. Thus, the constellation of state, professional and corporate logics standardizes service delivery and forms a barrier to patient participation.

Knowledge of how normative integration functions is sparse but necessary to ensure integrated health services [18, 19, 21]. Knowing how institutional logics materialize can help us understand why the achievement of patient participation and normative integration in the intervention becomes difficult. This does not mean that we expected to find the same logics in play at each level, as different actors have different responsibilities and perspectives, and as logics are context-dependent [21, 25]. However, institutional logics guide actors’ behavior and thus compete with the prescriptions of the intervention. This knowledge makes explicit the distance that exists between policy and practice and why it occurs: multiple goals and norms for practice are being produced through a range of institutional logics, which can hamper normative integration between levels of health services.

### Study limitations and strengths

Our research suffers from limitations that can be addressed in future research. Firstly, implementation of the intervention started only 6–12 months before our data collection. The results need to be interpreted as issuing from an early phase of implementation; the intervention may be carried out differently at a later time. The selection of rehabilitation wards is a potential source of bias because these wards may have a stronger focus on self-management than long-term wards, which could have made bureaucratic logics more salient. Hence, the results are not representative for other work tasks of service delivery; neither do they necessarily indicate the logics that dominate at each level more generally or capture variations in the field. Patients and health professionals could possibly have behaved more collaboratively because they were being observed [48]. Constructivist analyses assume multiple interpretations are possible, and other researchers could have interpreted the text differently [48]. However, the overall conclusions of our study are in line with those of other studies. The strength of the study is that it generates knowledge by being theoretically informed by what happens when this kind of interventions are implemented. Thus, this study provides transferable perspectives by pointing out how goals for an intervention differ between levels of health services. Moreover, that the logics we found to dominate are consistent with those reported in other studies [35, 53] suggests transferability to other patient groups with chronic diseases.

### Implications

This study illuminated differences in institutional logics and distance between policy goals and practices. There can be differences between policy goals and practices [15, 21], especially within integrated care contexts with network-based governance [21, 44]. In the present study, this entailed that actors themselves could develop intervention tools, and there were few formal regulations controlling their practices. This is an aspect for policy makers at the macro-level to consider. On the other hand, a central question that arises is whether the policy goals are too comprehensive to be carried out. There is a lack of evidence that ideals of integrated service delivery are implemented in contexts [13, 15]. The standardized approach is thought-provoking: it was the more bureaucratic logics that transcended levels. Hence, the extent to which the intervention made it possible for the older patients with multi-morbidity in this study to set the agenda for their individual service delivery was limited. To improve normative integration of the intervention, meso-level actors could perform user surveys to let patients assess the success of the intervention. Moreover, revision of the intervention tools so that they ask about several areas of patients’ lives and contain more detailed prescriptions for health professionals could counteract the fact that multiple normative justifications influence how the intervention is carried out.

## Conclusions

Normative integration was low within the intervention promoting patient participation in which older patients with multi-morbidity are encouraged to formulate individual goals for service delivery. Between the macro-, meso- and micro-levels of health services, values and actions were connected in different ways. Actors’ normative justifications for patient participation differed both within each of the institutional logics and in terms of the constellations of logics between the various levels of municipal health services. These findings broaden the understanding of how multiple set of norms co-exist and guide action; they also draw attention towards the dynamics between logics. We observed a reduction in the number of logics in play between the three levels. When patients were asked to formulate individual goals for service delivery, norms and goals for the intervention shifted from individualization to standardization between levels. Even though health professionals were engaging in the goal-setting intervention, the comparatively few distinct logics guiding their actions meant that service delivery was still centered more on what matters to the health services than on what matters to patients. Overall, the findings regarding how vertical normative integration contributes to patient participation were disappointing for the case being studied. Still, knowledge of institutional logics in services for patients with multi-morbidity provides a new theoretical frame that helps to understand why patient participation and integration of health services can be low. We hope that this line of sight will encourage further research on how institutional logics are reflected in professional work. More studies focusing on multiple institutional logics and multiple levels of health services could inform the normative integration which is necessary to integrated service delivery interventions.

## Supplementary Information

**Additional file 1.** Interview and observation guide

## Data Availability

The data generated and analyzed in the current study are not publicly available due to Norwegian privacy legislation and the form signed by the participants and municipalities about the study’s privacy. The data generated are available from the corresponding author on reasonable request.
