# Supplementary material for: ‘What matters to you?’ Normative integration of an intervention to promote participation of older patients with multi-morbidity – a qualitative case study
Source: BMC Health Serv Res. 2021 Feb 4;21:117. doi: 10.1186/s12913-021-06106-y (PMC7863321; doi:10.1186/s12913-021-06106-y)
Supplement: Supplementary file 1 — Additional file 1. Interview and observation guide [file 12913_2021_6106_MOESM1_ESM.docx]

# Additional file 1 – Interview and observation guides

## Interview guide focus groups

Can you describe what you do when you ask patients ‘What matters to you?’

Could you describe situations in which you asked: ‘What matters to you?’ and the patients’ answer had implications for the help you gave?

Could you describe situations in which you asked: ‘What matters to you?’ and the patients’ answer had minor implications for the help you gave?

Do patients participate differently than before you implemented the ‘What matters to you?’ procedure?

## Observation guide for meetings between health professionals and patients

This observation guide was filled out during and right after the meetings. The analysis of the meetings was however mostly based on the transcripts of audio recorded meetings, which allowed us to examine their normative justifications for patient participation according to the institutional logics.

| **THE MEETING** |  | RESEARCHER’S INTERPRETATION |
| --- | --- | --- |
| Time. | Time. Duration of the meeting. |  |
| Description of the place. | Where, how the place looked, what happened in the context. |  |
| Participants. | Number of persons, their roles.  Patient:  Health professionals:  Relatives: |  |
| Leader of the meeting |  |  |
| Structure of the meeting | How they carried out the meeting. |  |
| Agenda | The agenda for the meeting   - According to written documents/tools - According to what health professionals and/or patients said during the meeting |  |
| Division of tasks. |  |  |
| Did health professionals use tools or checklists? | Description of the tools and how they were used during the meeting. |  |
| **Interactions** |  |  |
| Did participants know each other? |  |  |
| Atmosphere |  |  |
| Formal/informal conversation | Areas of patients life and health they focused on. |  |
| Communication | Professional terminology used?  Interruptions?  Room for asking questions?  Nonverbal communication |  |
| **«What matters to you?»** | Who asked the question?  What happened? |  |
| What health professionals did to let patient participate. |  |  |
| What the patient did to participate. |  |  |
| Were there signs of less patient participation? |  |  |
| Assessment of how the researcher influenced the situation. | *The researcher in most of the meetings asked participants about their experience of being observed.* |  |
| Aspects which seems unclear, to be further examined (e.g. ask health professionals or patients after the meeting) |  |  |
| Emerging aspects in the meeting? |  |  |
